# Uncovering the regional localization of inhaled salmeterol retention in the lung

**DOI:** 10.1080/10717544.2018.1455762

**Published:** 2018-03-28

**Authors:** Erica Bäckström, Gregory Hamm, Anna Nilsson, Britt-Marie Fihn, Nicole Strittmatter, Per Andrén, Richard J. A. Goodwin, Markus Fridén

**Affiliations:** a Drug Metabolism and Pharmacokinetics, Respiratory, Inflammation and Autoimmunity IMED Biotech Unit, AstraZeneca, Gothenburg, Sweden;; b Pathology Sciences, Drug Safety & Metabolism IMED Biotech Unit, AstraZeneca, Cambridge, UK;; c Biomolecular Mass Spectrometry Imaging, National Resource for MSI, Science for Life Laboratory, Dept. of Pharmaceutical Biosciences, Uppsala University, Uppsala, Sweden;; d Translational PKPD Group, Department of Pharmaceutical Biosciences, Uppsala University, Uppsala, Sweden

**Keywords:** Pulmonary distribution, lung retention, mass spectrometry imaging, pharmacokinetics, inhalation

## Abstract

Treatment of respiratory disease with a drug delivered via inhalation is generally held as being beneficial as it provides direct access to the lung target site with a minimum systemic exposure. There is however only limited information of the regional localization of drug retention following inhalation. The aim of this study was to investigate the regional and histological localization of salmeterol retention in the lungs after inhalation and to compare it to systemic administration. Lung distribution of salmeterol delivered to rats via nebulization or intravenous (IV) injection was analyzed with high-resolution mass spectrometry imaging (MSI). Salmeterol was widely distributed in the entire section at 5 min after inhalation, by 15 min it was preferentially retained in bronchial tissue. Via a novel dual-isotope study, where salmeterol was delivered via inhalation and d_3_-salmeterol via IV to the same rat, could the effective gain in drug concentration associated with inhaled delivery relative to IV, expressed as a site-specific lung targeting factor, was 5-, 31-, and 45-fold for the alveolar region, bronchial sub-epithelium and epithelium, respectively. We anticipate that this MSI-based framework for quantifying regional and histological lung targeting by inhalation will accelerate discovery and development of local and more precise treatments of respiratory disease.

## Introduction

Effective management of respiratory diseases with inhaled corticosteroids (ICS) and bronchodilators demonstrate the utility of the inhaled route of delivery to directly access the target site while minimizing systemic drug exposure and side effects (Labiris & Dolovich, 2003a,b). Still, the complexity and heterogeneity of respiratory disease represent a major challenge for developing effective precision medicines (Pavord et al., 2018), and hence, besides anti-infectives, no new pharmacological classes of locally acting inhaled drugs have been introduced for many decades (Stein & Thiel, 2017). We hypothesize that an additional barrier to develop inhaled treatments is the lack of understanding of the spatial distribution of inhaled drugs. Without this information drug development programs are at risk of failing clinical trials for reasons related to the inadequate targeting of the diseased lung sub-structures.

Clinical imaging techniques including single photon emission computed tomography (SPECT) and gamma scintigraphy are unable to directly visualize biodistribution at required resolution (Dolovich & Labiris, 2004; Bäckman et al., 2018). These established analytical methodologies are inadequate as they lack the specificity to map the delicate and intertwined sub-structures and cell types of the lung. Furthermore, measurement of drug concentrations in plasma following inhalation can, at best, be used to interpret the overall absorption rate from lung to blood. In pre-clinical research, the primary means of studying inhalation pharmacokinetics (PK) is quantification of drug in blood and homogenized tissue but equally these data only supports conclusions around the rate of absorption – all spatial information is lost in the sample processing. Hence, up to this point, pre-clinical drug research has primarily focused on modulating physicochemical properties of the drug to achieve drug retention in the lung to provide a duration of pharmacological effect which is commensurate with once or twice daily dosing (Cooper et al., 2012). Further analysis has been practically impossible: manual dissection of alveolar versus main airway tissue is not feasible in rodents, broncho-alveolar lavage (BAL) provides no information on drug in tissue itself, tissue digestion and cell-fractionation risk compound de-localization, and quantitative auto-radiography requires labeled compound. This means that there is a significant analytical unmet need for researchers working in drug discovery of respiratory diseases.

Mass spectrometry imaging (MSI) is a technique that acquires molecular image data with high specificity and sensitivity while retaining spatial localization information (Caprioli et al., 1997). This enables mapping of the local distribution of multiplexed, label-free, molecular species in tissue sections at micro-meter scale (>5 µm). MSI has been previously applied to study the distribution of compounds in lungs but with limited spatial resolution (Nilsson et al., 2010; Marko-Varga et al., 2011; Prideaux et al., 2011; Sun et al., 2016; Matsumoto et al., 2017; Zecchi et al., 2013). MSI can also describe the morphology of lung based on the molecular profile of tissue sub-regions as alveoli, airways or blood vessels (Berry et al., 2011; Zemski Berry et al., 2017).

Salmeterol, a long-acting β_2_-adrenoreceptor agonist (LABA), displays a biphasic PK profile typical for bronchodilators (Cazzola et al., 2002). Concentration data from whole lung homogenates after inhalation of salmeterol show fast initial absorption followed by a slower absorption phase of the drug retained in the lungs. The regional localization has however never been established. The aim of this study was to investigate the regional (bronchial versus alveolar) and histological (epithelial versus sub-epithelial) sites of salmeterol retention in the lungs after inhalation and to compare the effects of route of administration on distribution using MSI. By gaining knowledge of the regional localization of the lung retention of a known bronchodilator, we are one step closer to being able to target inhaled drugs to specifically diseased lung sub-structures. This is critical for the development of safe and efficacious treatments.

## Methods and material

### Chemicals

Salmeterol was obtained from the AstraZeneca compound library and d_3_-salmeterol (98.2% chemical purity) from CDN Isotopes (Quebec, Canada). All other chemicals were of analytical grade and all solvents were of HPLC grade.

### Animals

Male Wistar-Han rats (Harlan, Horst, the Netherlands) weighing 300–400 g were housed in an Association for Assessment and Accreditation of Laboratory Animal Care (AAALAC)-accredited animal facility in groups of six individuals at 18–22 °C under a 12-h light/dark cycle with access to water and chow *ad libitum* for at least 5 days prior to the experiments. The study was approved by the Animal Ethics Committee of Gothenburg (134-2013).

### Drug administration and tissue collection

For administration via nebulization, salmeterol was formulated as a suspension (3 µm mass median diameter) in an aqueous vehicle containing DPPE-MPEG 2000, polysorbate 80 and a citric acid buffer. As with clinical pressurized metered dose inhaler (pMDI) products of water-soluble bronchodilators, the suspension formulation was required in this study for delivering enough drug material into the lung in very small volumes of droplet fluid. Jet nebulizer Swirler^®^ (AMICI, Spring City, PA) was used and the rats were dosed for 6 min to achieve a targeted lung deposited dose (LDD) of 700 µg/kg. The tail vein was used for intravenous (IV) administration, salmeterol and d_3_-salmeterol was formulated as a solution in 3% dimethylacetamide in 28% 2-Hydroxypropyl-β-cyclodextrin, and the dose was 7 mg/kg. In the rats receiving dual administration, salmeterol was first administered via nebulization immediately followed by the IV dose.

At the time of sampling were rats anesthetized with isoflurane (FORENE^®^, Abbott Scandinavia AB, Solna, Sweden) and a terminal blood sample was collected as previously described (Boger et al., 2015) to isolate plasma and stored at −20 °C until analysis. The heart and lung were removed from the thorax *en bloc*. Following removal of the heart the left lobe was dissected out, snap frozen in a horizontal position in dry ice-chilled isopentane and stored at −80 °C until sectioning. The remaining four lobes were stored at −20 °C until homogenization using Precellys bead-beating technology (Bertin technologies, Montigny le Bretonneux, France) as previously described (Bäckström et al., 2016b). The plasma and the homogenized lungs were analyzed by liquid chromatography-tandem mass spectrometry (LC-MS/MS) as previously described (Bäckström et al., 2016b).

### Sample preparation, MSI, and data analysis

Lungs were sectioned (10 µm) using a CM3050 cryo-microtome (Leica Biosystems, Nussloch, Germany) and thaw-mounted onto indium tin oxide coated glass slides (Bruker Daltonics, Bremen, Germany) for matrix-assisted laser desorption/ionization (MALDI) MSI or onto Superfrost slides (Fisher Scientific, Loughborough, UK) for desorption electrospray ionization (DESI) MSI and histological examination. Sectioning order and tissue position on slides were randomized in order to avoid any influence of sample preparation or conditions on MS measurement. Experimental blinding was not possible. Tissue section slides were stored at −80 °C until analysis. Hematoxylin and eosin (H&E) staining was performed on lung sections as previously described (Caldwell & Caprioli, 2005), imaged with Aperio CS2 digital pathology scanner (Aperio Tech., Oxford, UK), and visualized with ImageScope software (Aperio Tech.). MSI analysis of tissue sections were carried out using MALDI time-of-flight (TOF) (rapifleX, Bruker Daltonics) and DESI Q-Exactive (Thermo Fisher Scientific Inc., Bremen, Germany) mass spectrometers. For MALDI, lungs were coated with dihydroxybenzoic acid (DHB) MALDI matrix using the TM Sprayer (HTX Technologies, Chapel Hill, NC) as previously described (Swales et al., 2014). For high spatial resolution experiments, sublimation was used to apply DHB (Hankin et al., 2007). Images were collected at spatial resolutions 10–50 μm in positive detection mode over a mass range of 300–1000 Da. FleXcontrol 4.0 and flexImaging 4.0 (Bruker Daltonics) were used for MS parameter optimization and MSI experiment set up, respectively. Data management, analysis, and visualization were performed using SCiLS Lab MVS 2018a software (SCiLS GmbH, Bremen, Germany). MS images were normalized to the total ion count (TIC) to compensate for signal instabilities and allow multiple experiment comparison. Biological replicates (*n* = 2) were used for all experiment (time course) with at least two technical replicates per sample and shown in Supplemental Information. Exact masses measurements are extracted from high spectral resolution DESI-MSI dataset. Pearson correlation was used to compare the distribution of molecular markers to salmeterol related ions. Heterogeneity of salmeterol distribution was assessed by comparing pixel-to-pixel variation of salmeterol ions (IV versus inhaled) in whole lung (∼50,000 mass spectra) and relative standard deviation (r.s.d. in percentage) on mean was generated. Significant differences between samples were determined by using Student’s *t*-test (two sided) according to the normal distribution nature of dataset. A *p*-value < .05 was considered as significant. A bisecting k-means algorithm using a weak denoising and distance correlation as parameters was applied to provide unsupervised clustering of MSI data and corresponding segmentation map.

## Results

### Time course of inhaled salmeterol absorption and change in spatial distribution pattern

**Figure 1.**
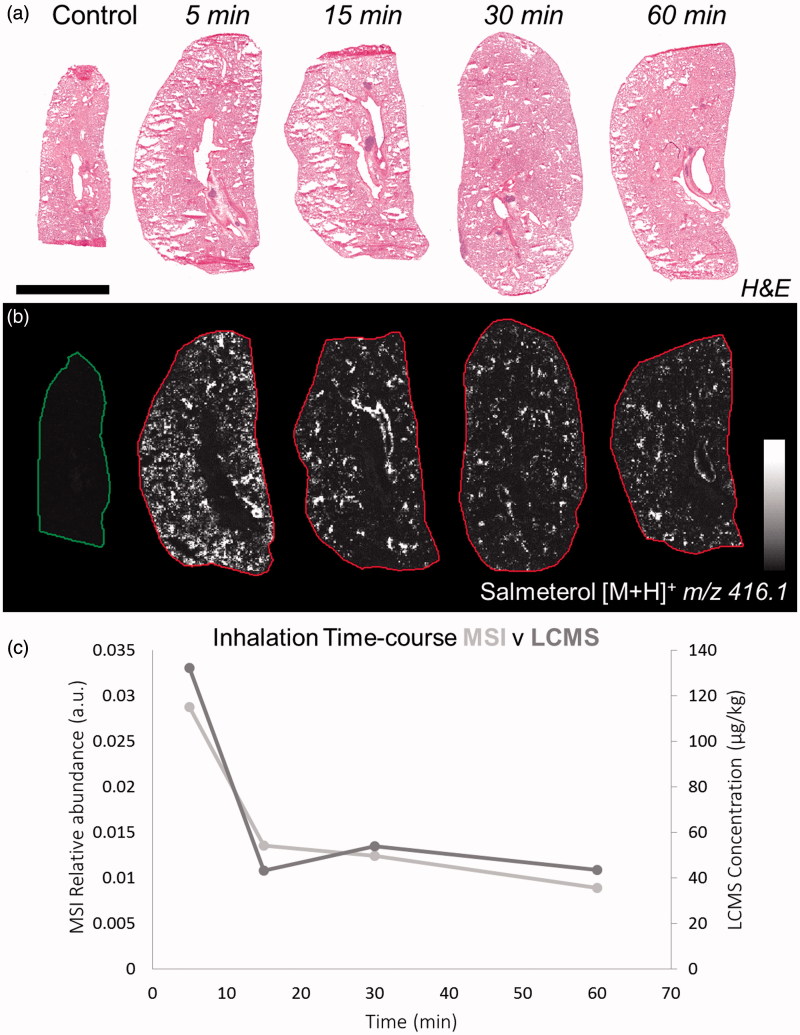
(a) H&E staining of dosed lung sections for morphological evaluation. (b) Distribution of salmeterol at 5, 15, 30, and 60 min after administration via nebulization using MALDI-MSI at 70 µm spatial resolution (duplicate biological replicates). White denotes the maximal signal intensity per image and black the minimal signal intensity observed. (c) Kinetic profile of salmeterol levels with overlay of its relative abundance from MSI (light grey) and its concentration from lung homogenate bioanalysis (dark grey). Intensity scale 0–100% Scale bar = 6 mm.

Salmeterol was identified based of its mass to charge ratio (*m*/*z*) from the protonated molecule (*m*/*z* 416.1, [M + H]^+^) in positive ionization mode (Supplementary Table S1). No detection of endogenous species was observed at the same *m*/*z* as salmeterol in control lung (between 416 and 416.2 Da). Lung sections were stained with H&E (Figure 1(a)) in order to match the morphology of the tissue with MS images from adjacent sections (Figure 1(b)). At the earliest time point, 5 min, salmeterol was evenly distributed between the alveolar and the bronchiolar regions whereas at 15 min and onwards it was retained in a less uniform distribution and appeared to co-localize with bronchioles (Figure 1(a,b) and Supplementary Figure S1). The MSI relative abundance of salmeterol from MS images correlated well with bioanalysis from tissue homogenates, both showing the typical bi-phasic profile for salmeterol (Figure 1(c)).

### Impact of administration route on salmeterol distribution

**Figure 2.**
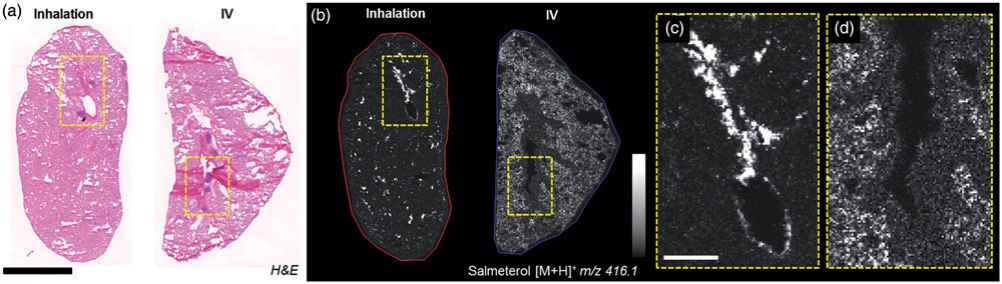
(a) H&E staining of inhaled and IV dosed lung sections. (b) MALDI images of corresponding tissues at 35 µm spatial resolution showing the differential distribution of salmeterol depending on administration route (duplicate biological and technical replicates). Inhaled salmeterol was preferably retained in bronchioles whereas systemic dose was more widely detected within tissue 30 min after delivery. (c and d) Higher magnification of a bronchiolar area in both conditions, the different administration routes clearly display differential distribution. Intensity scale 0–100%. Scale bars = 5 mm (black)/1 mm (white).

The distribution of salmeterol at 30 min after IV administration was extensive in the alveolar region, whereas after inhalation it was co-localized with the tissue of the main airways (Figure 2(a,b)). At a higher resolution, salmeterol clearly underlines epithelium of bronchioles in the inhaled lung (Figure 2(c)). Systemically administered salmeterol was mainly located in the alveolar region (Figure 2(d)).

### Dual-isotope administration study to quantify regional and histological lung targeting by inhalation

**Figure 3.**
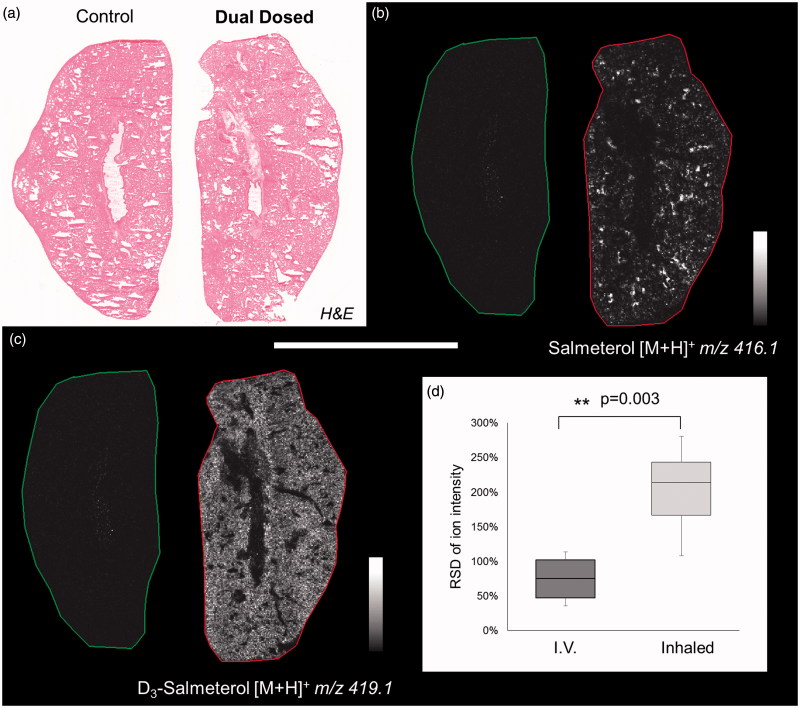
(a) H&E staining of control and dually dosed (salmeterol for inhalation and d_3_-salmetrol for IV) lung sections. (b and c) Salmeterol (inhaled) and d_3_-salmeterol (IV) distributions using MSI at 50 µm of spatial resolution showing the differential localization of both administered compounds (duplicate biological and triplicate technical replicates) at 30 min after delivery. Intensity scale 0–100% for both salmeterol versions. Scale bar = 1.2 mm. (d) Evaluation of salmeterol distribution heterogeneity depending on administration route (IV versus Inhaled) based on pixel-to-pixel relative standard deviation (RSD in %) in the whole lung image. A higher heterogeneity of the salmeterol signal is observed for inhaled compound than IV dosed (214% versus 75% in median, *n* = 6).

Dual-isotope administration of inhaled salmeterol and IV d_3_-salmeterol resulted in similar distribution patterns as after single dose administration within lung tissue (Figure 3(a)), i.e. highly localized for inhalation (Figure 3(b)) and more homogenous for IV (Figure 3(c)). To qualitatively evaluate the differential distribution of salmeterol, a statistical analysis of MSI data was performed. The pixel-to-pixel variation of salmeterol signal (relative standard deviation, RSD in %) shows a significant difference between the two ways of administrations with a higher heterogeneity for inhaled delivery than for IV (214% versus 75%, Figure 3(d)). Salmeterol isotopes were spatially correlated with markers of lung structures using Heme b (blood vessel marker), phospholipid PC (32:0) (alveolar marker) and phospholipid PC (36:4) (bronchiolar marker) identified based on their exact masses in Supplementary Table S1. The Pearson correlation coefficients indicated that inhaled salmeterol was correlated with bronchiolar marker whereas IV administered salmeterol was correlated with an alveolar marker (Table 1, Supplementary Figure S2). Cross-validation between DESI and MALDI confirmed good correlation in terms of salmeterol distribution (Supplementary Figure S3) and histological markers localization (Supplementary Figure S4).

**Table 1.** Spatial correlation between tissue endogenous marker (blood vessels, alveolar, and bronchiolar) and inhaled (salmeterol) and IV (d_3_-salmeterol) compound distributions.

	d_3_-Salmeterol	Salmeterol
Heme B, blood vessel marker	5%	0%
PC (32:0), alveolar maker	14%	0%
PC (36:4), bronchiolar marker	7%	10%
d_3_-Salmeterol	NA	2%
Salmeterol	2%	NA

Percentages are calculated based on Pearson correlation factor from the whole MS image of the lung (duplicate biological and triplicate technical replicates).

### High spatial resolution imaging and clustering analysis

**Figure 4.**
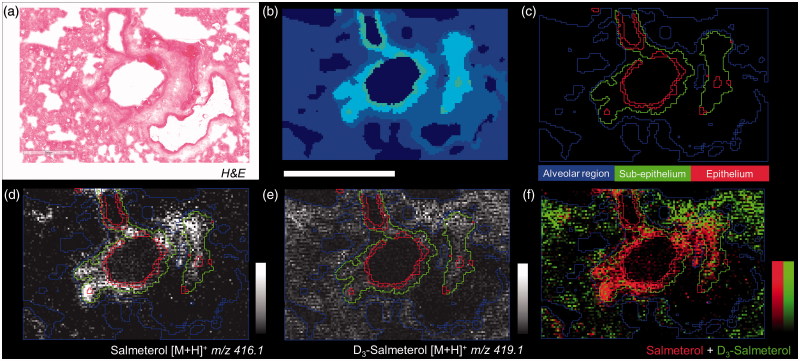
(a) H&E from dual dosed lung section focus on analyzed region. (b) Spatial clustering of high spatial resolution (10 µm) MSI of corresponding region showing similarities between tissue compartment molecular composition. (c) Region of Interest (ROI) generation from segmentation results corresponding to three tissue sub-structures (triplicate biological replicates). MS Images of inhaled (d), IV administered salmeterol (e), and overlay of both distributions (f). Data shows discrete localization of inhaled salmeterol to epithelium and sub-epithelium compared to IV dosed salmeterol within alveolar regions at 30 min after delivery. Intensity scale 0–100% for both salmeterol versions. Scale bar = 600 µm.

The histological structure of a tissue is reflected in its molecular composition which can be measured by MSI. Therefore, mass spectra spatial segmentation was applied to high spatial resolution images (10 µm) for allowing local salmeterol quantification in bronchiolar sub-structures. Groups of similar spectra were delineated on the tissue in a common color (Figure 4(a,b)), then regions of interest (ROI) were created following each segment providing quantitative information about molecular abundances (Figure 4(c)). Using non-supervised clustering it was seen that inhaled salmeterol correlated with sub-epithelial and epithelial segments (Figure 4(d,f)) whereas the systemically given salmeterol was homogeneously distributed in the alveolar segment (Figure 4(e,f)).

### Lung targeting factor calculation

**Figure 5.**
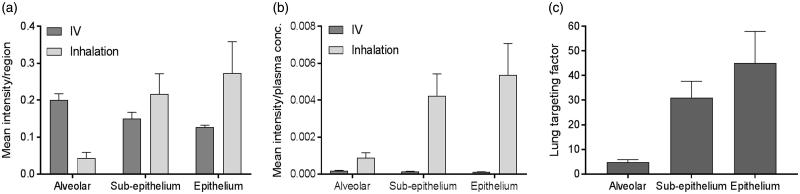
(a) Histogram of the mean intensity of salmeterol determined by MSI per segmented region, i.e. alveolar, sup-epithelium and epithelium, after inhalation or systemic dosing (*n* = 3) at 30 min after delivery. (b) The mean intensity in each region was normalized against the plasma concentration in respective administration group (*n* = 3). (c) The targeting factor is the ratio between inhalation and IV group in (b), in respective region, and it describes the gain with inhalation over systemic dosing at equivalent plasma concentrations.

The mean intensities of both salmeterol species were extracted in the alveoli, sub-epithelium and epithelium ROIs from the segmentation analysis (Figure 5(a) and Supplementary Figure S5). After normalization against respective plasma concentration the data clearly show that inhalation compared to IV, at similar plasma exposure, leads to higher distribution in all investigated regions of the lung (Figure 5(b)). The inhaled-to-IV ratio of normalized mean intensities (lung targeting factor) indicates that the inhaled route of delivery is associated with 5-fold higher targeting of the alveolar region as compared to IV administration, and 31- and 45-fold for the sub-epithelial and epithelial regions, respectively (Figure 5(c)). The lung targeting factor calculated from the mean intensity for the entire section, with no consideration of regions, was 10-fold (data not shown).

## Discussion

Local treatment of the lung via the inhaled route of administration revolutionized the treatment of asthma in the 1970s following the introduction of both selective β-agonist bronchodilators and ICS. The development continued with inhalation device advancement, longer-acting drug molecules, as well as the marketing of combinations products (Stein & Thiel, 2017). From a pharmaceutical perspective, development aims have remained focused on the treatment of the lung as a whole with emphasis on aspects like refined formulation (particle sizes) to better reach the peripheral regions of the lung (Leach et al., 2009). However, the development of new drugs for respiratory disease needs to move towards precision medicine (Pavord et al., 2018). This has increasingly highlighted that there are significant data- and knowledge-gaps in our understanding regarding drug distribution across lung sub-structures following inhalation. Such information is required for tailoring inhaled drug molecules and formulations to target the diseased sub-structures of the lung and/or avoid excessive exposure of those parts of the lung with no need for drug intervention. In the present study, we demonstrate for the first time the spatial distribution of an inhaled drug (salmeterol) showing selective lung sub-structural (bronchial) targeting using MSI. This was further appraised by simultaneous analysis of standard and deuterated salmeterol administered by different routes.

Our study describes the homogenous distribution of inhaled salmeterol in both the bronchiolar and alveolar region at the earliest time point (5 min), whereas from 15 min and onwards salmeterol was retained in a localized sporadic manner preferentially in the bronchioles (Figure 1(b)). Thus, the regional localization of salmeterol retention had been established. These findings add substance to a range of conceptual models of salmeterol retention and pulmonary selectivity. These models include the recognition that salmeterol has a large unbound drug volume of distribution in the lungs (*V*_u,lung_) and retention driven by lysosomal trapping as determined with a lung slice methodology (Bäckström et al., 2016a,b). Similarly, micro-kinetic models suggest that the retention of β-agonists is due to the interaction with the phospholipid membrane to produce a depot for maintenance of local efficacious drug concentrations around the target receptor (Sykes et al., 2014). The multiphasic lung profile of β-agonist bronchodilators (Figure 1(c)) has recently been characterized with a pharmacokinetic model structure with both a shallow (rapidly equilibrating) and deep (slowly equilibrating) lung compartment (Hendrickx et al., 2017) where the release of drug from the unidentified deep compartment provides the lung retention. In contrast, Borghardt et al. modeled another β-agonist, olodaterol, pharmacokinetics with three parallel pulmonary absorption processes, the slowest of which (*t*½ 21.8 h) was associated with 70% of the lung deposited dose and therefore suitable for once daily dosing (Borghardt et al., 2016). However accurately these models are describing inhalation PK data that is generated at the whole organ level, the histological/anatomical identity of the model compartments has remained nebulous. The experiments of the present study, however, strongly suggest that in order to provide accurate predictions of local exposure to inhaled drug it is required to use physiologically based pharmacokinetic (PBPK) models that include explicit and separate descriptions of tracheobronchial and alveolar lung regions (Bäckman et al., 2018).

Direct comparison of lung sections from different animals dosed via inhalation and IV was difficult, as no comparable structures could be found in the lung sections (Figure 2). In order to adequately address the impact of administration route, we performed a first-of-its-kind dual administration study with the delivery of an inhaled dose of salmeterol and a systemic dose of d_3_-salmeterol to the same animals. This allowed for direct comparison of the distribution and subsequently a deeper analysis of the observed heterogeneity following inhalation. The initial observation of heterogeneity following inhalation was confirmed by quantification of the pixel-to-pixel relative the standard deviation (RSD in %) in the whole lung section. For all scientific experiments involving animals the principle of the 3Rs, replacement, reduction, and refinement, are important considerations for the use of animals in drug testing. When performing this kind of dual administration study, we are using a method to directly reduce the number of animals used by obtaining more information from a single animal. Also, by combining the two administration routes in one animal, we bring the precision of comparison to a higher level and thus make better use the experimental animal. Technical considerations for the use of dual isotopes include having sufficient isotopic purity of the deuterated drug.

Unsupervised segmentation of lung tissue sections (Figure 4(b,c)) of dual-administered animals allowed unbiased quantification of inhaled versus IV administered drug in segments corresponding to bronchial epithelium, bronchial sub-epithelial tissue, and alveolar bed. Following normalization of segmental intensity with the measured plasma concentration of the respective salmeterol species (Figure 5(b)), it became evident the heterogeneity observed for inhalation effectively represented targeting of bronchial structures with drug levels in bronchial epithelium and sub-epithelium being respectively 45- and 31-fold higher than would be expected from systemic dosing. This lung targeting factor represents a means to express, for a defined location in the lung, the effective gain in drug concentration that is associated with inhaled delivery. Interestingly, the lung targeting factor for the rapidly perfused alveolar bed was also substantial (5-fold). The observation of having the highest exposure in the bronchial epithelium is not unexpected as these are the first cells to interact with the deposited drug. Additionally, the thickness of bronchial epithelial and sub-epithelial layers and the incomparably lower blood perfusion rate of bronchial versus pulmonary (alveolar) circulations are factors likely contributing to the observed bronchiolar retention. Hence, we hypothesize that the interactions of salmeterol with lung tissue involve non-specific binding and lysosomal uptake which occurs across all regions, and the release of which is slowest in the bronchial region results in the second absorption phase (Figure 1(b)) and a steady and durable presentation of free drug to the smooth muscle target cells. With the assumption of equivalent non-specific binding of inhaled and systemic salmeterol, it would be reasonable to expect that the 45-fold targeting factor also translates to an equivalent fold selectivity of bronchodilation over systemic side effects. This is however not proven in the present study.

Finally, the pattern of approximately equivalent concentrations in the epithelial versus sub-epithelial segments seems pharmacologically reasonable as the sub-epithelial space contains the smooth muscle target site. Other studies using auto-radiography of extremely lipid-soluble toxicants such as pyrene and benzo[a]pyrene in dog trachea have shown pronounced preferential distribution in the epithelium versus sub-epithelium (Gerde & Scott, 2001). This difference in epithelial versus sub-epithelial distribution is likely related to the physicochemical properties of the compounds and it illustrates the potential to modulate these by chemical design in order to target the (retention of) inhaled drug to the diseased regions or histological structures of the lung.

In conclusion, we have developed an experimental framework based on MSI of dual administration to determine with regional and histological resolution the effective gain in drug concentration (lung targeting factor) that is achieved by choosing the inhaled route of delivery. Using this methodology, we showed for the first time how inhaled salmeterol is selectively retained around its pharmacological target site in the sub-epithelium. By profiling chemically dissimilar inhaled drug molecules and the formulations thereof, we can now start exploring how the spatial distribution of inhaled drug can be modulated and optimized. This is a pre-requisite for inhalation therapy to transition from blindly treating the lung as a whole to precision inhaled medicine targeting the diseases lung sub-structures.

## Supplementary Material

IDRD_B_ckstr_m_et_al_Supplemental_Content.docx
