# Supplementary material for: Uncovering the regional localization of inhaled salmeterol retention in the lung
Source: Drug Deliv. 2018 Mar 28;25(1):838–45. doi: 10.1080/10717544.2018.1455762 (PMC6058612; doi:10.1080/10717544.2018.1455762)
Supplement: IDRD_B_ckstr_m_et_al_Supplemental_Content.docx [file IDRD_A_1455762_SM3922.docx]

**Supplemental information (SI)**

**Material and methods**

***DESI Imaging Parameters***

DESI MSI analysis was performed in positive ion mode and mass spectra were collected in the mass range of 300−1000 Da with a resolving power set to 70000 using a Q-Exactive (Thermo Fisher Scientific Inc.) mass spectrometer. The DESI ion source was operated at a spatial resolution of 100 µm on an automated 2D DESI source from Prosolia inc. (Indianapolis, IN, USA) with home built sprayer. A Methanol/water (95:5 v/v) mixture was used as the electrospray solvent at a flow rate of 1.5 μL/min and supply by a Dionex Ultimate 3000 LC pump (Thermo Fisher Scientific Inc.). Nebulizing nitrogen was used as gas at a pressure of 6.5 bar. The angle between the sprayer tip and the sample surface was set at 80° and the collection angle at 10°. The height distance between the DESI sprayer and the sample surface was set to 1 mm with the distance between the sprayer and the inlet capillary set to 7 mm. The distance between the sample surface and the inlet capillary of the mass spectrometer was <<1 mm. For line scan experiments, the parameters described above were used with a scan speed of 378.79 μm/s and an injection time set at 150 ms. Omnispray 2D (Prosolia) and Xcalibur (Thermo Fisher Scientific Inc.) software were used to set up MSI run. DESI MSI dataset consisted of individual horizontal line scans converted in mzml format using MSConvert and then combined into imzML file using the imzML converter V.1.3 ([www.maldi-msi.org](http://www.maldi-msi.org)).

**SI table 1**. Summary of species detected by MS imaging in lung sections. Identification was based on the m/z value measured during high spectral resolution DESI-MSI and compared to theoretical values of ion clusters (error expressed in ppm) using literature and metabolite databank matching. The molecular markers used for histological assessment of salmeterol localization were heme B and two phosphatidylcholine species, PC (32:0) and PC(36:4) describing blood vessels, alveolar and bronchiolar regions, respectively. *PC (total chain length : total degree of unsaturation).*

| Compound Name | Ion Cluster | *m/z calcd* | *m/z meas* | Error (ppm) | Tissue |
| --- | --- | --- | --- | --- | --- |
| Salmeterol | [M+H]^+^ | *416.2795* | *416.2788* | 1.7 | Inhalation |
| D_3_-Salmeterol | [M+H]^+^ | *419.2984* | *419.2987* | 0.7 | Intravenous |
| Heme B | [M]^+^ | *616.1768* | *616.1763* | 0.7 | Blood vessels marker |
| PC (32:0) | [M+K]^+^ | *772.5253* | *772.5234* | 2.5 | Alveolar marker |
| PC (36:4) | [M+K]^+^ | *820.5253* | *820.5241* | 1.5 | Bronchiole marker |

**SI figure captions**

**SI figure 1.** H&E stains images from lung samples of the time course study (vehicle and dosed at 5, 15, 30, and 60 min after administration via nebulisation with the animal number) for both biological replicates (duplicate biological replicates/time point) (left panel) and corresponding MS images of the inhaled salmeterol distribution from adjacent tissue sections at 70 µm spatial resolution (right panel). Intensity scale 0-100%. Scale bar=2 cm

**SI figure 2.** Pearson correlation coefficient representation for two molecular species. Relative abundances of an alveoli marker (from 0 to 65 arbitrary unit) is plotted against the relative abundance of I.V. dosed d_3_-salmeterol (from 0 to 10.5 arbitrary unit) for all pixels (red crosses) of a whole lung MS image (single biological replicate here). A correlation value is calculated based on linear regression generated and expressed in percentage, a value of 100% explain a perfect spatial correlation of two molecular species, 0% a complete anti-localization.

**SI figure 3.** Comparison of two salmeterol distributions using both imaging techniques validating the localisation of salmeterol. a) H&E staining images of dually administered and control lung samples (duplicate biological and triplicate technical replicatesfor dosed samples). b) MALDI-MS images (50 µm spatial resolution) of inhaled salmeterol, I.V dosed salmeterol and overlay of both distribution (salmeterol in red, D_3_-salmeterol in green). c) DESI-MS images on adjacent section showing the distribution of the same species as for MALDI experiment (100 µm of spatial resolution). Intensity scale 0-100% for both salmeterol versions and modalities. Scale bar=1.2 cm.

**SI figure 4.** Cross validation of histological marker localisation using two MS imaging modalities on same sample. a) H&E staining images of dually administered lung and control sample (duplicate biological and triplicate technical replicatesfor dosed samples). b) MALDI-MS images (50 µm spatial resolution) of molecular histological marker from alveoli (PC (32:0) in green), blood vessels (heme b in red,), bronchioles (PC (36:4) in blue) and overlay of the three distributions. c) DESI-MS images on adjacent section showing the distribution of the same species as for the MALDI MSI experiment (100 µm of spatial resolution). Intensity scale 0-100% for all markers and modalities. Scale bar=1.2 cm.

**SI figure 5.** High spatial resolution MALDI-MS images (10um spatial resolution) of control (n=2, left panel) and dosed (n=3, right panel) dually administered lung sections. a) Spatial segmentation images generated from MSI data (5 segments are reported from dark blue to light blue). b) Region of interest extracted from segmentation data, blue, alveolar region, green sub-epithelial layer and red epithelium. Both inhaled c) and I.V. d) dosed salmeterol distribution in corresponding measured region with the overlay of histological region of interest. Intensity scale 0-100% for both salmeterol versions. Scale bar=800 µm.
